# *Ustilago maydis* Secreted Endo-Xylanases Are Involved in Fungal Filamentation and Proliferation on and Inside Plants

**DOI:** 10.3390/jof7121081

**Published:** 2021-12-15

**Authors:** Ismael Moreno-Sánchez, María Dolores Pejenaute-Ochoa, Blanca Navarrete, Ramón R. Barrales, José I. Ibeas

**Affiliations:** Centro Andaluz de Biología del Desarrollo (CABD), Universidad Pablo de Olavide-CSIC-Junta de Andalucía, Ctra. Utrera km.1, 41013 Seville, Spain; imorsan@upo.es (I.M.-S.); lolapejenaute@hotmail.com (M.D.P.-O.); bnavrui@upo.es (B.N.)

**Keywords:** PCWDE, smut fungi, pathogenesis

## Abstract

Plant pathogenic fungi must be able to degrade host cell walls in order to penetrate and invade plant tissues. Among the plant cell wall degrading enzymes (PCWDEs) produced, xylanases are of special interest since its degradation target, xylan, is one of the main structural polysaccharides in plant cell walls. In the biotrophic fungus *Ustilago maydis*, attempts to characterize PCWDEs required for virulence have been unsuccessful, most likely due to functional redundancy. In previous high-throughput screening, we found one xylanase to be important for *U. maydis* infection. Here, we characterize the entire *U. maydis* endo-xylanase family, comprising two enzymes from the glycoside hydrolase (GH) 10 family, Xyn1 and Xyn2, one from GH11, Xyn11A, and one from GH43, Xyn3. We show that all endo-xylanases except Xyn3 are secreted and involved in infection in a non-redundant manner, suggesting different roles for each xylanase in this process. Taking a closer look inside the plant during the pathogenic process, we observed that all secreted xylanases were necessary for fungal proliferation. Finally, we found that at least Xyn11A accumulated in the apoplast of the infected plant after three days, highlighting the role of these enzymes as important secreted proteins during fungal proliferation inside plant tissues.

## 1. Introduction

Plant smut diseases, which mainly affect grasses, are caused by a variety of fungal species collectively known as smut fungi. These pathogens are able to produce large amounts of teliospores, mainly in host floral organs, affecting their reproduction [1]. Smut diseases result in the loss of important crop cultivars, including wheat, barley, and maize, among others [2]. The biotrophic pathogen *Ustilago maydis*, currently one of the most well-studied smut fungi, is an exception to the pattern of floral teliospore formation. *U. maydis* is able to colonize all aerial parts of its host, the maize plant, inducing plant tumors in the last stages of colonization, where teliospores develop [3,4]. The pathogenic cycle occurs when two sexually compatible strains come into contact on the maize plant surface and pheromones activate mutually compatible receptors. Then, they form a conjugation tube and mate, developing a filamentous cell cycle-arrested dikaryon [5,6]. These filaments sense physical and chemical plant signals, generating a morphogenetic structure called the appressoria, which mediates fungal penetration into plants [7]. After penetration, cell cycle arrest is released and the fungus expands into a branched filamentous form that induces hypertrophied plant cells, macroscopically visible as tumors [3,8,9,10,11]. With the onset of plant tumor formation, diploid cells are formed after fusion of nuclei. Finally, hyphae subdivide as individual cells and undergo teliospore development.

The initial phases of fungal infection include the formation of infective structures in response to topographical (e.g., stomatal pores), chemical (e.g., epicuticular waxes), or physical (e.g., hydrophobicity) plant cues. Many pathogenic biotrophs use a combination of turgor pressure exerted by the infective structure and the secretion of plant cell wall-degrading enzymes (PCWDEs) to breach cell walls without affecting host cell viability [12,13,14]. PCWDEs are particularly important for phytopathogenic fungi that do not have specialized penetration structures, although all of them require these enzymes during the late stages of plant invasion [15]. To protect themselves against cell wall degradation and fungal infection, host plants produce PCWDE inhibitors (e.g., pectinase inhibitors are common in dicots and noncommelinoid monocots, while xylan-degrading enzyme inhibitors are common in crops and grasses) [15]. 

Although *U. maydis* uses the appressoria to penetrate the plant surface, in comparison with appressoria produced in other rust and pathogen fungi, it shows reduced size, a lack of melanization, and a thin cell wall, suggesting that this structure does not produce penetration by mechanical force [16,17,18,19], but rather by secreting degrading enzymes. In fact, it has been described that *U. maydis* PCWDEs are differentially regulated during maize infection [13,20,21]. Since the plant cell wall is primarily composed of cellulose microfibrils embedded in a matrix of pectin, hemicellulose, lignin, and structural proteins [15], the most promising putative *U. maydis* PCWDE candidates are catalytic enzymes involved in cellulose degradation (exo-cellulases, endo-β-1,4-glucanases, and β-glucosidases), hemicellulose degradation (endo-β-1,4-xylanases, endo-β-1,4-glucanases, β-xylosidases, β-mannases, arabinoxylanases, and arabinofuranosidases), and pectin degradation (polygalacturonases, pectin lyases and pectate lyases). Of these, 32 PCWDEs have been shown to be differentially regulated during maize colonization: 3 cellulases, 24 hemicellulases, and 5 pectin-degrading enzymes [21]. However, attempts to demonstrate a role for PCWDEs in *U. maydis* virulence have been unsuccessful, most likely due to gene redundancy. In fact, single deletions of the three genes encoding α-L-arabinofuranosidases *afg1*, *afg2*, and *afg3* did not considerably affect virulence [20,22], although a triple deletion strain did show reduced penetration efficiency with a significant decrease in virulence capacity [20]. However, this triple deletion strain was still able to penetrate the maize plant surface, colonize, and develop tumors. It has also been reported that simultaneous deletion of three *U. maydis* pectinase genes did not impair virulence [23]. 

Xylanases constitute another family of PCWDEs that are differentially regulated during *U. maydis* infection. Xylanases hydrolyze the 1,4-β-d-xylosidic linkages in xylan, the principal component of hemicellulose. Xylan is one of the main structural polysaccharides in plant cell walls and the second most abundant in nature [24]. It shows a complex branched structure with a core backbone chain made of 1,4-linked β-d-xylopyranosyl units decorated with different side-chain groups (e.g., glucuronopyranosyl, α-l-arabinofuranosyl). The side groups used vary between different plant sources [25]. This complexity makes the xylanases a heterogeneous family, and many microorganisms produce several xylanases [24]. Most xylanases are secreted enzymes, as the large size of xylan makes its cellular uptake difficult [26]. However, some intracellular xylanases are responsible for further degradation of xylo-oligomers released from extracellular degradation [27,28,29]. 

Despite their heterogeneity, xylanases share sequence and domain similarities that allow their classification into different groups. In agreement with the CAZy database, a broadly used classification system for glycosyl hydrolases [30], functional endoxylanases can be found in glycosyl hydrolase (GH) families 5, 7, 8, 10, 11, and 43, with the majority found in GH10 and GH11 [24,31]. GH10 xylanases typically have a high molecular weight and a low pI, compared to the low molecular weight and high pI of the GH11 family [24]. In addition, GH10 xylanases are less selective than GH11 ones and are able to degrade any decorated form of xylan [32]. The abundance of xylan and its importance for plant cell walls make it likely that secreted xylanases are important enzymes for penetration and/or invasion into the plant by pathogenic fungi. In agreement with this, different plants produce xylanase inhibitors, such as *Triticum aestivum* xylanase inhibitor (TAXI), xylanase inhibitor protein (XIP), and thaumatin-like xylanase inhibitor (TLXI) [33]. However, the role of fungal xylanases in plant pathogenesis is still poorly understood. Although deletion of the gene encoding a GH11 xylanase in the necrotrophic fungi *Botrytis cinerea* has been shown to produce a drastic reduction in infection capability [34], and more recently a study on the necrotrophic fungus *Rhizoctonia cerealis* revealed a role in infection for a GH10 xylanase [35], deletion of different xylanase genes in the hemi-biotrophic fungi *Fusarium oxisporum* and *Magnaporthe oryzae* has shown no effect on plant infection [36,37,38], probably due to the redundancy between different enzymes in the family. 

In a recent screen for *U. maydis* virulence factors, we found the xylanase Xyn1 to be important for infection [22]. In addition to Xyn1, we found three additional endo-xylanases in the genome of *U. maydis*. Taking advantage of this genetically tractable model organism, we wanted to address whether all other xylanases of this biotrophic fungus had a role in infection and possible redundancy. We observed that three out of the four *U. maydis* xylanases were secreted and that single, double, and triple deletions of the genes encoding for them reduced infection symptoms to a similar degree, discarding functional redundancy between different xylanases. To get better insight into the possible role of these secreted xylanases during infection, we studied the different steps of the infection process and found that secreted endo-xylanases are involved in proper filamentation and fungal progression on the plant surface and inside the plant, suggesting different roles for these enzymes during the pathogenic process in addition to plant penetration. Furthermore, we detected the presence of at least one xylanase in the apoplast of infected plants (three days after inoculation), supporting their importance as secreted proteins required for fungal progression inside plant tissues.

## 2. Materials and Methods

### 2.1. Strains, Plasmids, and Growth Conditions

*Escherichia coli* DH5α was used for cloning with plasmids pGEM-T easy (Promega, Madison, WI, USA) and pJET1.2/blunt (ThermoFisher Scientific, Carlsbad, CA, USA). Growth conditions for *E. coli* have been described previously [39].

All *U. maydis* strains used in this study are listed in Table 1. As previously described [40], *U. maydis* pre-cultures and cultures were performed in YEPSL (0.4% bactopeptone, 1% yeast extract, and 0.4% saccharose) unless otherwise specified. 

Cell wall integrity, ER, and oxidative stress assays were carried out with cultures grown at 28 °C to exponential phase in complete media (CM) supplemented with 2% D-glucose (CMD) and spotted at 0.4 OD_600_ onto CM plates supplemented with calcofluor white (CFW) 10 or 40 µg/mL (Sigma-Aldrich, St. Louis, MO, USA), Congo Red 10 or 50 µg/mL (Sigma-Aldrich, St. Louis, MO, USA), 4 mM DTT (iNtRON Biotechnology, Seongnam, Gyeonggi, ROK), tunicamycin 1 µg/mL (Sigma-Aldrich, St. Louis, MO, USA), sorbitol 1 M (Sigma-Aldrich, St. Louis, MO, USA), 2% DMSO (Sigma-Aldrich, St. Louis, MO, USA), H_2_O_2_ 1.5 mM (Sigma-Aldrich, St. Louis, MO, USA), NaCl 1 M (Sigma-Aldrich, St. Louis, MO, USA), and 0.005% SDS (Sigma-Aldrich, St. Louis, MO, USA). Plates were incubated for 48 h at 28 °C. 

For conjugation tube formation assay, FB1 single xylanase deletion mutants were grown to exponential phase in liquid CMD. Then, cells were diluted to 0.5 OD_600_ in 1 mL and incubated in a wheel at room temperature for 5 h with 1 µL (2.5 mg/mL) of pheromone a2. As a control, cells were also incubated with 1 µL of DMSO, since the pheromone is diluted in this organic solvent.

For mating assays, cells were grown in liquid YEPSL until exponential phase, washed twice with sterile bi-distilled water, spotted onto PD-charcoal plates, and grown for 24–48 h at 25–28 °C.

Maize (*Zea mays*) infection assays were performed as previously described [19], with minor modifications. Briefly, *U. maydis* cells were grown at 28 °C to exponential phase in liquid YEPSL and concentrated to an OD_600_ of 3, washed twice in water, and injected into seven-day-old maize seedlings (Early Golden Bantam). Disease symptoms were quantified at 14 days post-infection. Statistical analyses were performed in GraphPad Prism 6 software.

### 2.2. Molecular Biology and Genetics Methods

Molecular biology techniques were used as described by Sambrook, Frisch, and Maniatis in *Molecular Cloning: A Laboratory Manual* [39]. *U. maydis* DNA isolation and transformation were carried out following the protocol described by Schulz et al. [44].

Xylanase deletion mutants were generated by homologous recombination as described previously [22]. Primers used in this study are listed in Supplementary Table S1.

To perform the double and triple xylanase mutant deletion, Δ*xyn1*::nat construct from pJET Δ*xyn1*::nat was amplified by PCR with Q5 DNA polymerase (New England Biolabs, Ipswich, MA, USA) using primers xyn1KO5-1/xyn1KO3-2 and integrated into CL13 Δ*xyn2*, generating CL13 Δ*xyn1*Δ*xyn2* strain. Then, Δ*xyn11A*::hyg construct from pJET Δ*xyn11A*::hyg was amplified by PCR with Q5 DNA polymerase (New England Biolabs, Ipswich, MA, USA) using primers xyn11AKO5-1/xyn11AKO3-2 and integrated into CL13 Δ*xyn1*Δ*xyn2*, generating CL13 Δ*xyn1*Δ*xyn2*Δ*xyn11A* strain.

For complementation of the ∆*xyn1* mutant, the sequence upstream of *xyn1* ORF until the next gene containing approximately 1 kb that would correspond to its promoter was amplified by PCR Q5 DNA polymerase (New England Biolabs, Ipswich, MA, USA) using primers Pxyn1_fwd/XmaI_Pxyn1_rev, adding a *XmaI* restriction site. After digestion with *XmaI* restriction enzyme (New England Biolabs, Ipswich, MA, USA), *xyn1* promoter was cloned into p123 P*_otef_*:*xyn1* (containing *xyn1* ORF under the control of *otef* promoter) previously digested with *XmaI* and *PvuII* restriction enzymes, removing the *otef* promoter and generating the plasmid p123 P*xyn1*:*xyn1*. This plasmid was *SspI* digested, purified with MEGAquick-spin™ Plus Total Fragment DNA Purification Kit (iNtRON Biotechnology, Seongnam, Gyeonggi, ROK), and integrated into the *ip*-locus of CL13 Δ*xyn1* protoplasts. 

For Δ*xyn2* and Δ*xyn11A* complementation, the *xyn2 and xyn11A* genes were amplified by PCR using Q5 DNA polymerase (New England Biolabs, Ipswich, MA, USA) and primers designed in the NEBuilder assembly tool (Pxyn2_fwd/Pxyn2_rev for *xyn2* and Pxyn11A_fwd/Pxyn11A_rev for *xyn11A*). All fragments were cloned into p123 plasmid previously digested with *PvuII* and *NotI* using NEBuilder^®^ HiFi DNA Assembly (New England Biolabs, Ipswich, MA, USA) and ligation transformed into *E. coli* competent cells. p123 P*_xyn2_*:*xyn2* and p123 P*_xyn11A_*:*xyn11A* derivative plasmids were digested with *SalI* and *SspI*, respectively. Both digested plasmids were purified with MEGAquick-spin™ Plus Total Fragment DNA Purification Kit (iNtRON Biotechnology, Seongnam, Gyeonggi, ROK) and integrated into the *ip*-locus of CL13 Δ*xyn2* and CL13 Δ*xyn11A* protoplasts, respectively. 

For GFP tagging of *xyn2*, *xyn11A*, and *xyn3*, gene ORF was amplified by PCR using Q5 DNA polymerase (New England Biolabs, Ipswich, MA, USA) and primers with *BamHI* and *NcoI* restriction sites to clone ORFs in frame with GFP into p123 GFP plasmid [45] previously digested with *BamHI* and *NcoI*. T4 DNA ligase was employed to ligate both fragments, and ligation transformed into *E. coli* competent cells. Every p123 derivative plasmid was *SspI* digested and transformed into *U. maydis* SG200 wild-type protoplasts.

To obtain CL13 2xRFP Δ*xyn1*, p123 2xRFP plasmid was *SspI* digested, purified with MEGAquick-spin™ Plus Total Fragment DNA Purification Kit (iNtRON Biotechnology, Seongnam, Gyeonggi, ROK), and integrated into the *ip*-locus of CL13 Δ*xyn1* protoplasts.

For the CL13 2xRFP Δ*xyn2* and CL13 2xRFP Δ*xyn11A* strains, deletion construction was PCR amplified using Q5 DNA polymerase (New England Biolabs, Ipswich, MA, USA) and transformed into CL13 2xRFP protoplasts. 

For the Xyn2 and Xyn11A mCherry-HA fused proteins, gene ORFs were PCR amplified with primers containing *SacII* and *NcoI* restriction sites and RSIATA sequence to clone them in frame into a p123 derivative plasmid harboring mCherry-HA under the control of *pit2* promoter [43]. Both digested and purified p123 P*_pit2_*:mCherry-HA and gene ORF were ligated in a 1:5 vector:fragment proportion with T4 DNA ligase and transformed in *E. coli* competent cells. Subsequent *U. maydis* transformation after *SspI* plasmid digestion was performed into the *ip*-locus of SG200 protoplasts.

Since the *xyn1* ORF contains an *NcoI* restriction site inside its sequence, clonation into p123 P*_pit2_*:mCherry-HA was performed following the same strategy described above, but using *XbaI* restriction enzyme instead of *NcoI* for gene ORF and plasmid.

For relative fungal biomass quantification, maize seedlings were infected with *U. maydis*, and 3- and 8-days post infection, leaves were ground in liquid nitrogen. Total DNA was isolated with DNeasy Plant Mini Kit (Qiagen, Hilden, Germany) according to the manufacturer’s instructions. Fungal biomass was then quantified by qRT-PCR using a Real-Time CFX Connect (Bio-Rad, Hercules, CA, USA) and SYBR^®^ Premix Ex Taq™ II (Tli RNase H Plus) (Takara Bio INC, Kusatsu, Japan) according to the manufacturer’s protocol, measuring the signal of *ppi1* fungal gene relative to the plant gene *gapdh*. For this process, 0.6 and 6 ng of total DNA was used as template for *gapdh* and *ppi1* reaction, respectively. 

### 2.3. Sequence Alignment and Phylogenetic Analysis

BlastP was used to search for xylanase sequences in other organisms. The alignments were obtained using MAFFT v7. Phylogenetic analysis of xylanases was inferred by using the maximum likelihood method based on the JTT matrix-based model [46]. The G-INS-iterative strategy (recommended for <200 sequences with global homology) was used to generate GH11 and GH43 alignment and the L-INS-iterative strategy (recommended for <200 sequences with one conserved domain) to generate GH10 alignment. Refined datasets were applied to remove redundant sequences, and missing data were eliminated. MaxAlign was selected to maximizing the size of gap-free columns. Initial trees for the heuristic search were obtained automatically by applying the neighbor-joining algorithm. Trees were visualized and annotated using Interactive Tree of Life (iTOL v6, https://itol.embl.de/).

The unrooted phylogenetic tree (Figure S1) was built with xylanases from *U. maydis*, *U. hordei*, *U. bromivora*, *U. trichophora*, *Pseudozyma hubeiensis*, *Pseudozyma brasiliensis*, *Sporisorium reilianum*, *S. scitamineum*, *Anthracocystis flocculosa*, *Botrytis cinerea*, *Magnaporthe oryzae*, *Trichoderma reesei*, *Aspergillus niger*, *Aspergillus nidulans*, *Fusarium oxysporum*, *Fusarium graminearum*, *Puccinia striiformis* f. sp. *tritici*, *Puccinia graminis* f. sp. *tritici*, *Blumeria graminis*, *Verticillium dahliae*, and *Saccharomyces pastorianus*. The tree was generated automatically by applying the neighbor-joining algorithm and the Jukes–Cantor genetic distance model to the 126 selected xylanase sequences previously aligned by Geneious alignment (Geneious Prime 1 February 2019).

### 2.4. Infection Stage Analysis

For on plant filamentation and appressoria quantification, wild-type cells harboring cytoplasmic CFP and mutant cells harboring cytoplasmic RFP were grown in liquid YEPSL until exponential phase, washed twice with bi-distilled water, and concentrated to an OD_600_ of 3. Cells from wild-type and mutant strains were mixed in equal proportions, centrifuged, and concentrated to an OD_600_ of 3. Maize seedlings were infected, and leaves were recovered 18–20 h post-infection. Infected leaves were stained with calcofluor white (Sigma-Aldrich, St. Louis, MO, USA) to visualize *U. maydis* filaments and appressoria in a fluorescence microscope.

To analyze maize plant colonization capability, *U. maydis* cultures were grown at 28 °C to exponential phase in liquid YEPSL and concentrated to an OD_600_ of 3, washed twice in water, and injected into seven-day-old maize seedlings. Three days post-infection, leaves were de-stained with ethanol for at least 24 h, treated at 60 °C with 10% KOH for 4 h, washed four times in phosphate buffer, and then stained with propidium iodide (PI) to visualize plant tissues in red and wheat germ agglutinin (WGA)-AF488 to visualize the fungus in green for 30 min in a vacuum pump with 5 min vacuum and 5 min rest cycles. At least four leaves from two independent experiments were stained and visualized by fluorescence microscopy (Leica SPE DM2500, Leica, Wetzlar, Germany).

### 2.5. Protein and Blotting Assays

For colony secretion assay, cells were grown in YEPSL to an OD_600_ of 0.6–0.8, then were spotted onto a nitrocellulose filter secured to a rich media plate. After drop-drying, plates were sealed with parafilm and incubated face-up at 28 °C for 16 h. Cells growing on the nitrocellulose membrane were washed with distilled water, using a roller to eliminate all cells, and the membrane was incubated with mouse polyclonal anti-GFP antibody (Roche, Mannheim, BW, Germany) (1:1000). As secondary antibody, anti-mouse IgG-horseradish peroxidase conjugated antibody (1:5000; Sigma-Aldrich, St. Louis, MO, USA) was used. Immunoreactive dots were developed by SuperSignal™ West Femto Maximum Sensitivity substrate (ThermoFisher Scientific, Carlsbad, CA, USA). Image gel and membrane acquisition was carried out with ChemiDoc XRS (Bio-Rad, Hercules, CA, USA).

To determine whether xylanases were secreted to the maize apoplasts, cells harboring xylanases tagged with mCherry under the control of *pit2* promoter [43] were grown in liquid YEPSL until exponential phase, washed twice in water, and concentrated to an OD_600_ of 3. Around 200 maize seedlings for each strain were infected, and 3 dpi leaves were recovered to isolate apoplastic fluid by vacuum infiltration. For that purpose, 8 cm of leaf from 1 cm below the infection site was cut and coated with bi-distilled water in a big beaker with a steel sieve on top. Three cycles of 15 min vacuum at 60 mbar and 2.5 min atm pause were applied in a vacuum chamber while the water was continuously stirred with a magnetic stir bar to remove air bubbles. Then, leaves were carefully dried with paper towels, placed in syringes inside 50 mL tubes, and centrifuged at 3000 rpm for 15 min at 4 °C. Apoplastic fluid was pooled and stored at −80 °C or directly precipitated with chilled acetone and mCherry blotted using mouse polyclonal anti-mCherry antibody (Roche, Mannheim, BW, Germany) (1:1000). The same secondary antibody and blot development conditions described above were used here.

Southern blots of mutants for xylanases were performed with 60 µg of DNA from each *U. maydis* strain and 50–80 ng of plasmids containing each xylanase deletion cassette (pJET Δ*xyn1*::nat, pJET Δ*xyn2*::gen, and pJET Δ*xyn11A*::hyg). Purified DNA was digested with *EcoRI* restriction enzyme for nourseothricin (Nat) resistant strains or *HindIII* for geneticin (Gen) and hygromycin (Hyg) resistant strains. Plasmid pJET Δ*xyn2*::gen contains two sites for *HindIII* restriction enzyme, while plasmids pJET Δ*xyn1*::nat and pJET Δ*xyn11A*::hyg contain a single restriction site for *EcoRI* and *HindIII*, respectively. Digested DNA samples were separated in 0.8% agarose gel, then depurined in 0.25 M HCl, washed with distilled water, denatured with solution 1 (1.5 M NaCl, 0.5 M NaOH), neutralized with solution 2 (1 M Tris, 1.5 M NaCl pH 7.4), and transferred to a Whatman Hybond-N^+^ nitrocellulose membrane (Sigma-Aldrich, St. Louis, MO, USA). After UV-light crosslinking with 70 mJ/cm^2^, the membrane was hybridized with the corresponding probe for each resistance marker, which was previously labelled with digoxigenin (DIG) using a PCR DIG Probe Synthesis Kit (Roche, Mannheim, BW, Germany) following the manufacturer’s protocol and primers indicated in Supplementary Table S1. Membrane was then washed twice with solution 3 (30 mM sodium citrate pH 7.0, 0.3 M NaCl, 0.1% SDS), twice with solution 4 (1.5 mM sodium citrate pH 7.0, 15 mM NaCl, 0.1% SDS), and once with solution 5 (100 mM malic acid pH 7.5, 150 mM NaCl) and then incubated with 1% blocking reagent (Sigma-Aldrich, St. Louis, MO, USA) and with primary mouse anti-DIG antibody (1:1000). After being washed twice with solution 6 (0.3% Tween20, 100 mM malic acid pH 7.5, 150 mM NaCl), blot detection was performed with the chemiluminescent alkaline phosphatase substrate CSPD (Roche, Mannheim, BW, Germany) (1:3000). Membrane acquisition was carried out with ChemiDoc XRS (Bio-Rad).

### 2.6. Microscopy

For visualization of DNA content and cellular morphology, cells were stained with DAPI and observed by differential interference contrast (DIC) and fluorescence microscopy using a DeltaVision microscopy system comprising an Olympus IX71 microscope (Olympus, Shinjuku, Tokyo, Japan) and CoolSnap HQ camera (Photometrics, Tucson, AZ, USA).

For in plant quantification of filament and appressoria formation in co-infection experiments with *U. maydis* CFP and RFP labelled strains, 20 hpi leaf samples were stained with calcofluor white (Sigma-Aldrich, St. Louis, MO, USA) to visualize fungal material and then checked for CFP or RFP fluorescence in the DeltaVision microscopy system.

To analyze the *U. maydis* progression inside the maize plant, leaf samples stained with PI and WGA-AF488 (described above) were examined using a Leica SPE (DM2500, Leica, Wetzlar, Germany) confocal microscope.

Image processing was carried out using Adobe Photoshop CS5 (Adobe, San Jose, CA, USA) and ImageJ (public domain).

## 3. Results and Discussion

### 3.1. Ustilago maydis Xylanases Are Secreted and Required for Full Virulence

We have previously shown that protein glycosylation is essential for *U. maydis* virulence, since mutants for genes in the two main glycosylation pathways are completely avirulent [47,48]. In a screen for *U. maydis* virulence factors related to glycosylation, we found UMAG_04422 (now called Xyn1) encoding an endo-1,4-β-xylanase (EC 3.2.1.8) in the GH10 family, which is involved in plant infection [22]. In order to identify other *U. maydis* xylanases, we performed a BlastP search against the *U. maydis* genome using the Xyn1 sequence as input, finding UMAG_03411 (now called Xyn2), which also belongs to the GH10 family. As xylanases are also found in the GH 5, 7, 8, 10, 11, and 43 families, we performed an analysis for known proteins from those families in the *U. maydis* genome by examining the MycoCosm database [49]. In this fungal genomics database, we found four annotated xylanases: the two GH10 xylanases previously shown (Xyn1 and Xyn2), the GH11 protein UMAG_06350 (UmXyn11A) annotated as an endo-1,4-beta xylanase, and the GH43 protein UMAG_04897 (now called Xyn3), an uncharacterized protein related to endo-1,4-beta-xylanase in *U. trichophora*. Among them, UmXyn11A has been previously identified and functionally characterized as an endo-1,4-β-xylanase capable of degrading xylan [50,51]. 

**Figure 1:**
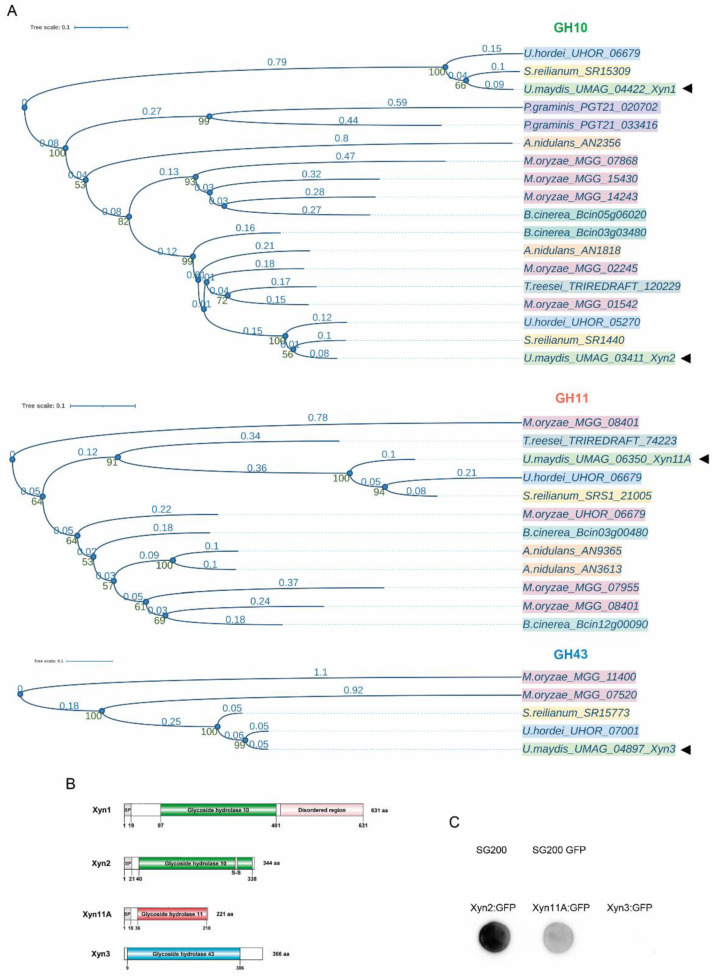
*Ustilago maydis* possesses four xylanases: two from the GH10 family, Xyn1 and Xyn2, a GH11 member, Xyn11A, and Xyn3 from the GH43 family. (**A**) Phylogenetic study of GH10, GH11, and GH43 xylanases across fungi. Each species is represented by a different color: *U. maydis* (light green), *U. hordei* (blue), *S. reilianum* (yellow), *P. graminis* (purple), *M. oryzae* (pink), *B. cinerea* (dark green), *A. nidulans* (orange), *T. reesei* (gray). Xylanases from *U. maydis* Xyn1, Xyn2, Xyn11A, and Xyn3 are indicated with arrowheads. Glycoside hydrolase (GH) domains are also color-coded: GH10 (green), GH11 (orange), and GH43 (blue). Bootstraps are indicated in green numbers down the nodes and distances are indicated in blue above each branch. (**B**) Schematic representation of four xylanases identified in *U. maydis*. GH domains of each xylanase are indicated, and signal peptide in N-terminal region is indicated by SP. (**C**) Secretion of Xyn2, Xyn11A, and Xyn3 (tagged with GFP) was assayed in a colony secretion assay. Wild-type SG200 strain was used as control for proper colony washing, and SG200 cells expressing cytoplasmic GFP under control of constitutive *otef* promoter served as cell lysis control (SG200-GFP).

To get a better picture of the possible peculiarities of the xylanase family of *U. maydis*, we performed a phylogenetic analysis of all xylanases found in other selected fungal phytopathogens, including closely related Basidiomycetes such as *U. hordei* and *Sporisorium reilianum*, the more distant rust fungus *Puccinia graminis*, and the Ascomycetes *Magnaporthe oryzae*, *Trichoderma reesei*, *Aspergillus nidulans*, and *Botrytis cinerea* (Figure 1A). We observed that Xyn1 from smut fungi is very distant from most of the other fungal GH10 xylanases, including smut fungus Xyn2 (Figure 1A and Figure S1). The sequence analysis shows that Xyn1 contains a disordered region of 200 amino acids in its C-terminal domain (Figure 1B), which is only present in the other two more divergent GH10 xylanases in this phylogenetic analysis: UHOR_06909 and sr15309 (from *U. hordei* and *S. reilianum*, respectively). This intrinsically disordered region (IDR) has been proposed to facilitate posttranslational modifications or protein–protein interactions or to regulate protein half-life during infection [52,53]. It is also remarkable that, in contrast to the high number of GH10 and GH11 xylanases, only a few GH43 xylanases are found in either Basidiomycetes or Ascomycetes (Figure 1A and Figure S1).

We wanted to know which of the identified xylanases are secreted and thus could potentially have a role in plant infection. Xyn1 has been previously identified as a secreted glycoprotein downstream of glycosidase I (Gls1) [22]. We performed a colony secretion assay to analyze whether xylanases Xyn2, Xyn11A, and Xyn3 were also secreted. As expected, given the presence of signal peptides in their N-terminal regions, Xyn2 and Xyn11A, but not Xyn3, were found to be secreted (Figure 1B,C). Thus, we excluded Xyn3 from further analysis since it would be expected to have a role in xylan-derivative carbohydrate metabolism inside fungal cells (under study) rather than in extracellular xylan degradation, as has been recently described in *Neurospora crassa* [54].

**Figure 2:**
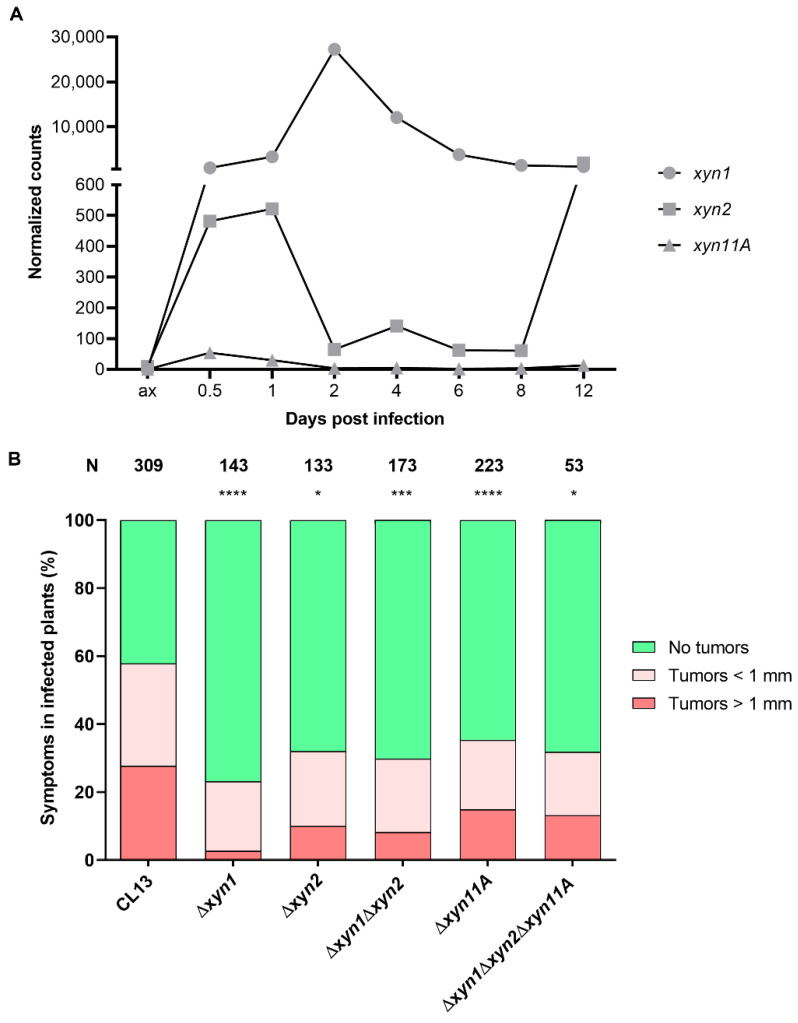
*Ustilago maydis* xylanases are differentially expressed during infection and are required for full virulence. (**A**) *xyn1*, *xyn2*m and *xyn11A* expression levels represented as normalized counts obtained from high-throughput transcriptomic analysis of *U. maydis* during pathogenesis [21]. (**B**) Quantification of symptoms for plants infected with indicated strains 14 days post infection. Total number of infected plants is indicated above each column. At least three biological replicates were analyzed. Mann–Whitney statistical test was performed for each mutant versus corresponding wild-type strain (* *p*-value < 0.05; *** *p*-value < 0.005; **** *p*-value < 0.001).

In order to decipher whether these secreted xylanases are expressed during the infection process, we analyzed their expression during maize plant infection at different time points using data from a high-throughput transcriptomic analysis of *U. maydis* pathogenesis [21]. These data show that *xyn1* was the most highly expressed xylanase during infection, with maximum expression at 2 dpi. *xyn2* had a much lower expression level, with a first peak during the first hours of infection and another one at a later stage (12 dpi). Finally, *xyn11A* was faintly expressed during the infection process, with higher expression at 0.5 dpi (Figure 2A). These data suggest that Xyn1 may be the main xylanase operating during biotrophic development, perhaps assisted by the other two xylanases at different stages of infection. 

To examine this possibility, we analyzed the virulence capability of single, double, and triple secreted xylanase deletion mutants. Significantly, the loss of any of the three xylanases resulted in virulence defects. As can be observed in Figure 2B, Figures S2 and S3, all single mutants showed reduced virulence in different genetic backgrounds. All mutants were checked by Southern blot analysis and complemented by re-introduction of the endogenous locus (Figure S2). Surprisingly, the double Δ*xyn1*Δ*xyn2* and the triple Δ*xyn1*Δ*xyn2*Δ*xyn11A* mutants showed nearly the same effect as the single ones (Figure 2B). This epistatic effect suggests that all xylanases have a non-redundant role during pathogenesis, and in agreement with the expression patterns, they may act at different stages of infection. 

The lack of fully non-virulent PCWDE mutants has commonly been explained by redundancy and compensatory effects between PCWDEs of the same family [15,35,55,56,57,58,59]. Although the quadruple deletion mutant, including the non-secreted Xyn3, was not created in this study, it is highly improbable that this mutant would result in an avirulent phenotype. Thus, our data suggest that such redundancy may also occur between PCWDEs with different catalytic activities, not just among xylanases themselves. Specifically, complete xylan hydrolysis requires additional enzymes. Although endo-1,4-β-d-xylanases are responsible for cleavage of the backbone chain, side group degradation is catalyzed by other enzymes, including α-l-arabinofuranosidases and α-d-glucuronidases [24]. Thus, it would be interesting to test the redundancy between cooperating enzymes for xylan degradation to further confirm their requirement for pathogenesis.

### 3.2. Xylanases Are Necessary to Assure Proper Fungal Filamentation and Progression Inside the Plant 

**Figure 3:**
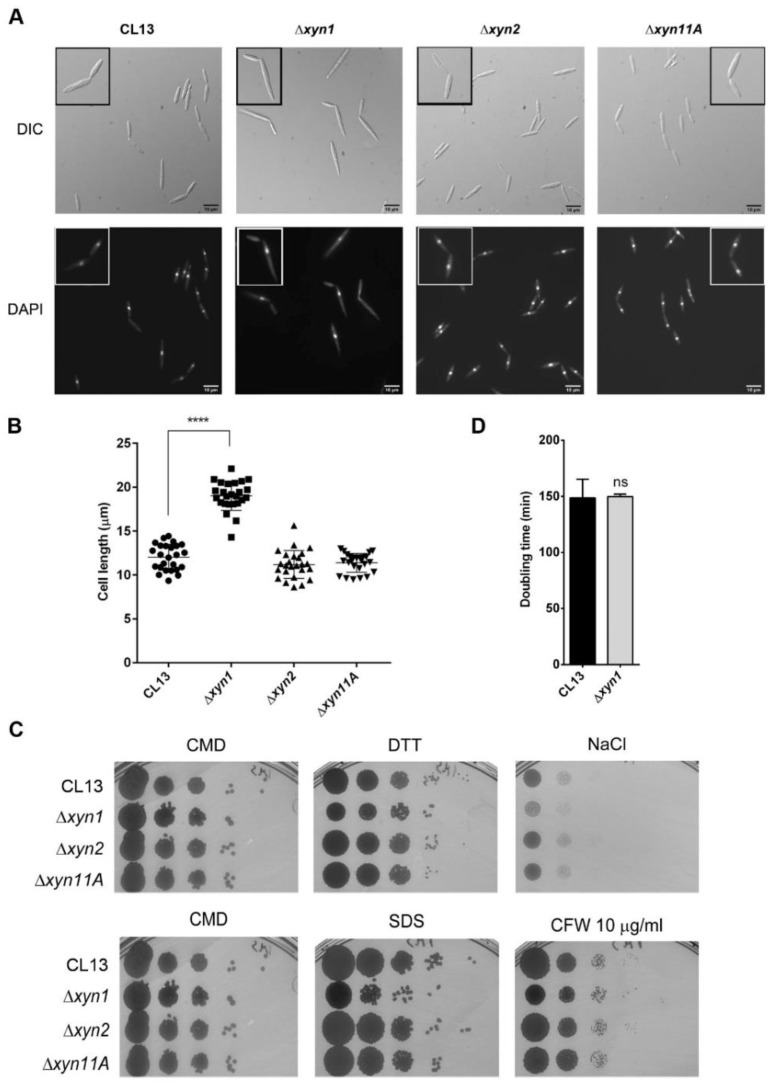
Effects of xylanase gene deletion on axenic growth. (**A**) Nuclei of exponential phase cells stained with DAPI. Scale bars represent 10 µm. (**B**) Length of CL13 wild-type, Δ*xyn1*,Δ*xyn2* and Δ*xyn11A* strains was measured in rich media culture at exponential phase. Quantification was carried out for 25 cells. T-test statistical analysis was performed (**** *p*-value < 0.001). (**C**) Spot tests to assay ER stress (DTT), osmotic stress (NaCl), cell membrane and oxidative stress (SDS), and cell wall integrity (CFW). Rich media (CMD) without drug was used as growth control. (**D**) Doubling time was calculated from growth curve performed in triplicate using rich media. T-test statistical analysis was performed (ns, not statistically significant).

To discard general cellular defects in the deletion mutants, which could explain the observed defects in the infection process, we analyzed growth alterations, endoplasmic reticulum (ER), osmotic and oxidative stress resistance, and cell wall integrity under axenic conditions. DTT and tunicamycin were used as ER stressors [60], sorbitol and NaCl as osmotic stressors, H_2_O_2_ as oxidant [61], calcofluor white (CFW) and Congo red as cell wall integrity sensors [62], and SDS as membrane-perturbing drug [62]. We found no significant differences in any of the conditions assayed, except for a subtle reduction in growth of *xyn1* deletion mutant on all of the compounds tested but tunicamycin, where the opposite effect was observed (Figure 3 and Figure S4). Strikingly, the lack of *xyn1* resulted in longer cells (Figure 3A,B), which was consistent among independent mutants with single Δ*xyn1* cassette integrations in two different genetic backgrounds (Figure S5). It is notable that this phenotype indicates a role for Xyn1 in the fungal cell itself, which suggests that this secreted xylanase may also have xylanase-independent activity, as fungal cells lack xylan. Despite the cells being longer and slightly more susceptible to some stressors, the doubling time of this mutant was similar to the wild-type strain (Figure 3D). All together, these data point to a specific role for these enzymes in plan pathogenesis rather than a pleiotropic effect indirectly affecting infection. 

**Figure 4:**
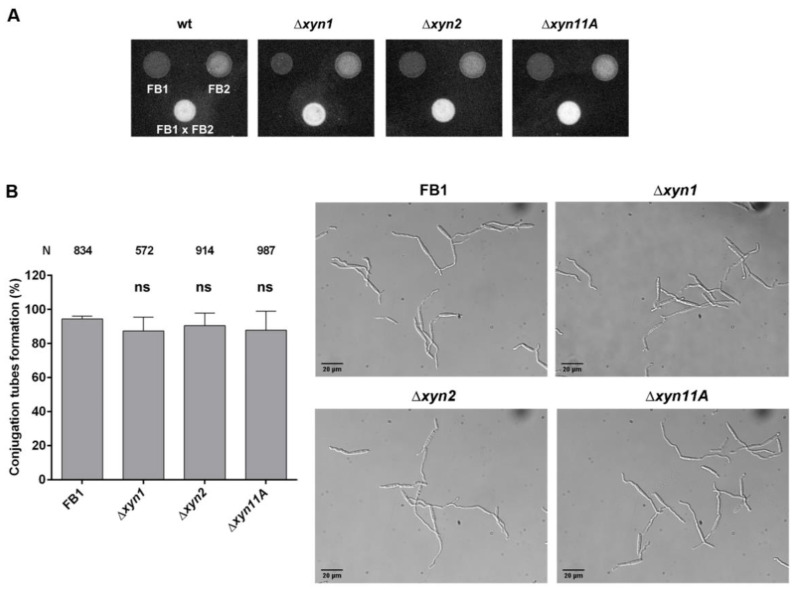
Mating and conjugation tube formation are unaffected by xylanase gene deletion. (**A**) Mating assay between compatible *U. maydis* FB1 and FB2 wild-type and Δ*xyn1*, Δ*xyn2*, and Δ*xyn11A* strains on PD-charcoal (PD-Ch) plates. (**B**) Conjugation tube formation for FB1 wild-type and xylanase deletion mutants was quantified after 5 h incubation with *a2* pheromone in axenic conditions. Scale bar represents 20 µm. Total number of cells, corresponding to four biological replicates, is indicated above each column. T-test statistical analysis was performed (ns, not statistically significant).

To determine which step of pathogenic development is affected by the loss of xylanases, we first examined the mating capability between sexually compatible cells. We observed similar mating efficiency and conjugation tube formation in all xylanase deletion mutants compared to the wild-type strains (Figure 4A,B). Therefore, pathogenic defects that affect xylanase mutant infection probably occur during host interactions. 

**Figure 5:**
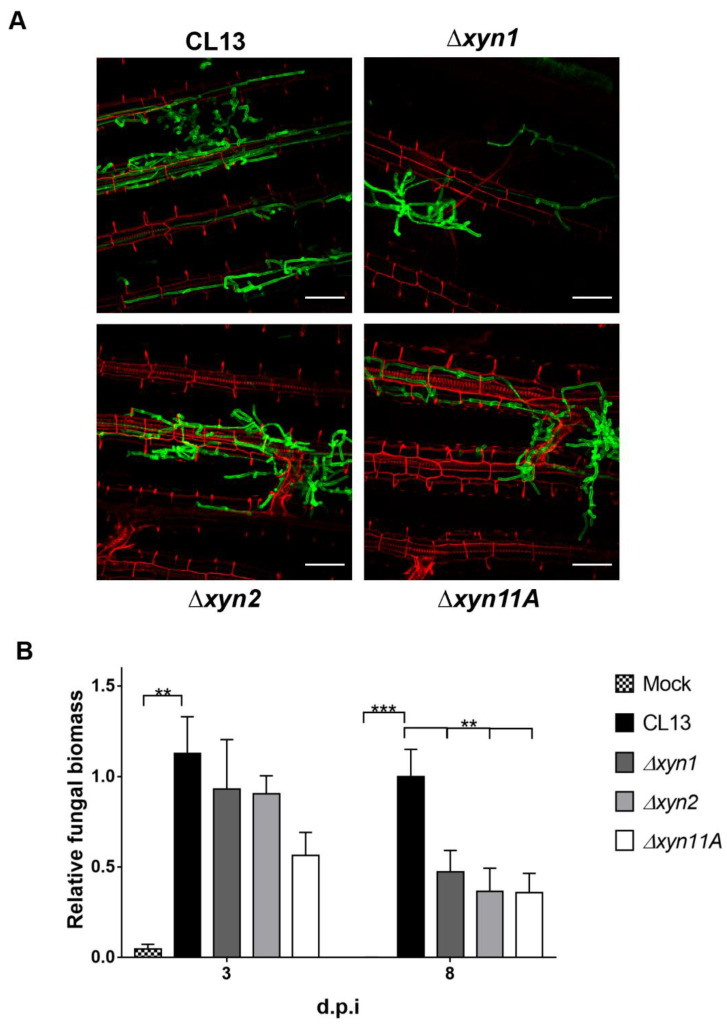
Fungal progression inside plant is impaired in the absence of xylanases. (**A**) Maize leaves from plants infected with CL13, CL13 Δ*xyn1*, CL13 Δ*xyn2*, and CL13 Δ*xyn11A* strains at 3 dpi were stained with propidium iodide (red) and *U. maydis* hyphae with WGA-AF-488 (green) and visualized by fluorescence microscopy. Scale bar represents 43 µm. (**B**) Fungal relative biomass in maize plants infected with indicated strains or water as mock treatment was quantified by qPCR. Four biological replicates were analyzed. T-test statistical analysis was performed (** *p*-value < 0.01; *** *p*-value < 0.001).

To address whether fungal colonization inside plant tissue was impaired, we visualized infected maize leaves at three days post-infection by microscopy and quantified fungal biomass inside the plant by performing qPCR of DNA samples in infected maize leaves at three- and eight-days post-inoculation (dpi). Although all xylanase mutants were able to perform cell-to-cell progression inside the plant with normal morphology (Figure 5A), we observed defective proliferation for all mutants inside the plant, with a significant reduction in detected biomass at 8 dpi but not at 3 dpi (Figure 5B). 

**Figure 6:**
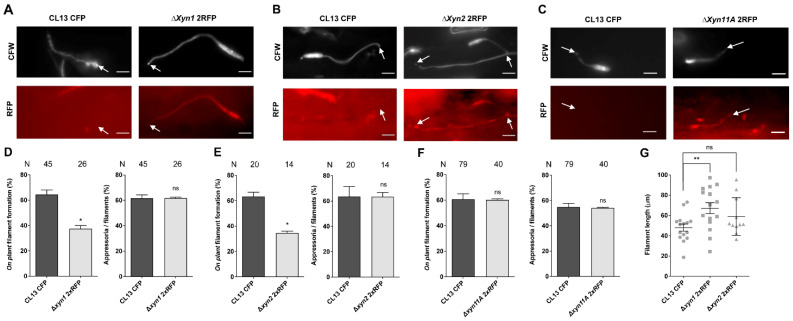
Filamentation on plant surface is reduced in the absence of Xyn1 and Xyn2. Maize seedlings were co-infected with CL13 CFP and CL13 2xRFP (**A**,**D**,**G**) Δ*xyn1*, (**B**,**E**,**G**) Δ*xyn2***,** or (**C**,**F**) Δ*xyn11A* mutant strains. After 18 h, infected leaves were stained with calcofluor white (CFW), and formation of filaments and appressoria was measured by scoring RFP fluorescence. Representative images of normally developed appressoria are shown in (**A**–**C**). Filament and appressoria efficiency are shown in (**D**–**F**). Length of filaments with appressoria is quantified in (**G**). White arrows indicate appressoria. Scale bar represents 10 µm. At least two leaves from two independent experiments were analyzed. T-test statistical analysis was performed (ns, not statistically significant; * *p*-value < 0.05; ** *p*-value < 0.01).

The reduced fungal progression of xylanase mutants inside the plant together with the induction of expression of these genes during the biotrophic establishment stage suggest that *U. maydis* proliferation inside plant tissues requires degradation of xylan in cell walls. However, we wanted to further investigate a possible additional role for xylanases in earlier stages of infection, when they start to be expressed, such as filamentation on the plant surface and appressoria formation. Although appressoria formation was not affected, we observed a significant reduction in filamentation capability on the plant surface for both ∆*xyn1* and ∆*xyn2* mutants versus the wild-type strain (Figure 6A,B,D,E), with longer filaments formed (Figure 6G). These defects were not observed in the ∆*xyn11A* mutant (Figure 6C,F), suggesting a more specific role of this xylanase in later stages of infection.

Many pathogenic fungi use the combination of turgor pressure and secreted cell wall degrading enzymes to penetrate plant tissues [12,13,63,64,65]. Once inside the plant, the fungi must continue degrading the cell walls in order to progress from cell to cell. In this scenario, we can postulate that *U. maydis* uses xylanases to degrade plant cell walls and proliferate inside the plant. However, the alteration in the number and length of filaments on the plant surface observed for ∆*xyn1* and ∆*xyn2* mutants is surprising. One possibility is that *U. maydis* secretes xylanases during the filamentation process and the residue resulting from xylan degradation acts as a signal for fungal penetration. In this way, the fungus might be able to identify regions where plant cell walls are more exposed as potential penetration sites. Once inside the plant, *U. maydis* would continue producing xylanases to degrade the cell walls, allowing proper proliferation of hyphae. In our studies we have not been able to identify different stages of the infection process affected by all xylanases, thus further investigations are required to know why these xylanases show non-redundant roles. 

### 3.3. Xyn11A Is Secreted to the Apoplast during Fungal Progression Inside the Plant

**Figure 7:**
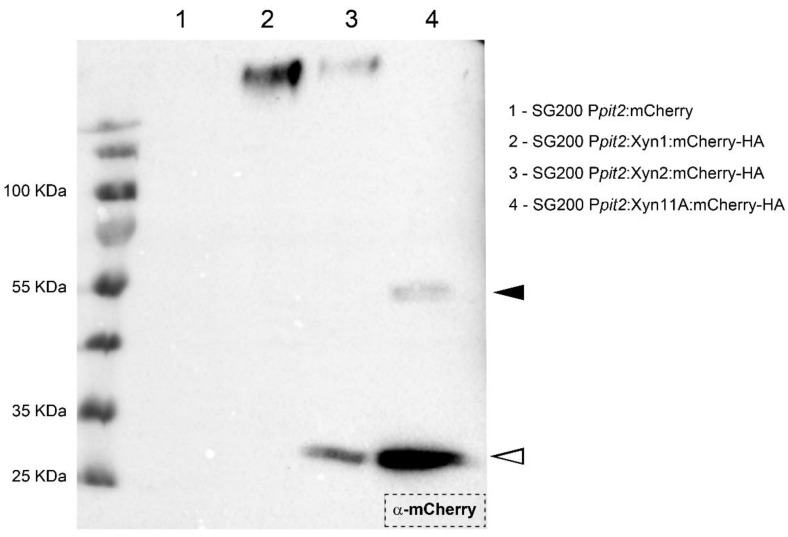
Xyn11A is secreted into maize apoplast. Apoplastic fluid was isolated 3 days post-inoculation from maize leaves infected with SG200 strains harboring Xyn1:mCherry-HA (2), Xyn2:mCherry-HA (3), or Xyn11A:mCherry-HA (4) under the control of *pit2* promoter [43,66]. Cytoplasmic mCherry under the control of *pit2* promoter in SG200 background (lane 1) was used as a control for cell lysis. Black arrowhead indicates 51 KDa band corresponding to Xyn11A:mCherry-HA and white arrowhead indicates 28.8 KDa band corresponding to free mCherry.

Given that xylanases are secreted in vitro and are required for fungal progression inside the maize plant, we wanted to determine if they are secreted into the maize apoplast during infection. To test this, we infected plants with SG200 strains harboring Xyn1-, Xyn2-, or Xyn11A:mCherry-HA fusion proteins under the control of *pit2* promoter, which is induced during the first days of infection [43], and purified apoplastic fluid at three days post-infection. The results obtained by anti-mCherry Western blot showed a band near 55 KDa corresponding to Xyn11A:mCherry-HA (51 KDa) and a second band between 25 KDa and 35 KDa equivalent to free mCherry (28.8 KDa) (Figure 7). To our knowledge, this is the first time this enzyme has been identified in the apoplast of infected plants. This observation strongly supports our previous results showing a role for xylanases in fungal progression inside the plant. In contrast, western blots for Xyn1 and Xyn2 in apoplast extracts resulted in the detection of bands corresponding to unexpectedly high molecular weights. Thus, it remains to be determined whether or not Xyn1 and Xyn2 are also secreted into the maize apoplast during *U. maydis* infection.

## 4. Conclusions

Here, we show that xylanases secreted by *U. maydis* GH10 and GH11 are involved in filament formation on the plant surface as well as during hyphae progression inside maize plant cells. Our results indicate that at least xylanase 11A is secreted into the maize apoplast during infection, which suggests that it has an important role in fungal progression inside the plant. Interestingly, the epistatic effect observed here for the different secreted xylanase mutants suggests that there is not such a high degree of redundancy in *U. maydis*, as has been observed for other fungi. Instead, each secreted xylanase should have an independent role during infection, and further investigations are required to identify these roles. In addition, the lack of completely non-virulent phenotypes in the triple xylanase deletion mutant suggests that these enzymes may act in conjunction with other types of PCWDEs to ensure successful xylan degradation and full pathogenic development. In the future, it would be of interest to determine what other PCWDEs might cooperate with the xylanases in the infection process or, conversely, whether *U. maydis* infection does not in fact primarily depend on PCWDEs.

## Figures and Tables

**Table 1:** Strains used in this study.

Strain	Relevant Genotype	Reference
FB1	a1 b1	[41]
FB2	a2 b2	[41]
FB1 Δ*xyn1*	a1 b1 Δ*xyn1*::nat	[22]
FB2 Δ*xyn1*	a2 b2 Δ*xyn1*::nat	[22]
FB1 Δ*xyn2*	a1 b1 Δ*xyn2*::gen	This work
FB2 Δ*xyn2*	a2 b2 Δ*xyn2*::gen	This work
FB1 Δ*xyn11A*	a1 b1 Δ*xyn11A*::hyg	This work
FB2 Δ*xyn11A*	a2 b2 Δ*xyn11A*::hyg	This work
SG200	a1 mfa2 bW2 bE1	[42]
SG200 3xGFP	a1 mfa2 bW2 bE1 P*_otef_*:3xGFP:cbx	[22]
SG200 *xyn2*:GFP	a1 mfa2 bW2 bE1 P*_otef_*:*xyn2*:GFP:cbx	This work
SG200 *xyn11A*:GFP	a1 mfa2 bW2 bE1 P*_otef_*:*xyn11A*:GFP:cbx	This work
SG200 *xyn3*:GFP	a1 mfa2 bW2 bE1 P*_otef_*:*xyn3*:GFP:cbx	This work
SG200 P*_pit2_*:mcherry-HA	a1 mfa2 bW2 bE1 P*_pit2_*:mcherry-HA:cbx	[43]
SG200 P*_pit2_*:*xyn1*:mcherry-HA	a1 mfa2 bW2 bE1 P*_pit2_*:*xyn1*:mcherry-HA:cbx	This work
SG200 P*_pit2_*:*xyn2*:mcherry-HA	a1 mfa2 bW2 bE1 P*_pit2_*:*xyn2*:mcherry-HA:cbx	This work
SG200 P*_pit2_*:*xyn11A*:mcherry-HA	a1 mfa2 bW2 bE1 P*_pit2_*:*xyn11A*:mcherry-HA:cbx	This work
CL13	a1 bE1 bW2	[42]
CL13 Δ*xyn1*	a1 bE1 bW2 Δ*xyn1*::nat	[22]
CL13 Δ*xyn2*	a1 bE1 bW2 Δ*xyn2*::gen	This work
CL13 Δ*xyn1*Δ*xyn2*	a1 bE1 bW2 Δ*xyn1*::nat Δ*xyn2*::gen	This work
CL13 Δ*xyn11A*	a1 bE1 bW2 Δ*xyn11A*::hyg	This work
CL13 Δ*xyn1*Δ*xyn2*Δ*xyn11A*	a1 bE1 bW2 Δ*xyn1*::nat Δ*xyn2*::gen Δ*xyn11A*::hyg	This work
CL13 Δ*xyn1* P_xyn1_:*xyn1*	a1 bE1 bW2 Δ*xyn1*:gen P_xyn1_:*xyn1*:cbx	This work
CL13 Δ*xyn2* P_xyn2_:*xyn2*	a1 bE1 bW2 Δ*xyn2*:gen P_xyn2_:xyn2:cbx	This work
CL13 Δ*xyn11A* P_xyn11A_:*xyn11A*	a1 bE1 bW2 Δ*xyn11A*:hyg P_xyn11A_:*xyn11A*:cbx	This work
CL13 CFP	a1 bE1 bW2 P*_otef_*:CFP:cbx	This work
CL13 2xRFP Δ*xyn1*	a1 bE1 bW2 P*_otef_*:2xRFP:cbx Δ*xyn1*::nat	This work
CL13 2xRFP Δ*xyn2*	a1 bE1 bW2 P*_otef_*:2xRFP:cbx Δ*xyn2*::gen	This work
CL13 2xRFP Δ*xyn11A*	a1 bE1 bW2 P*_otef_*:2xRFP:cbx Δ*xyn11A*::hyg	This work

## Supplementary Materials

The following are available online at https://www.mdpi.com/article/10.3390/jof7121081/s1. Figure S1: Fungal xylanase phylogenetic tree; Figure S2: Xylanase mutants contain a single cassette integration, leading to similar virulence defects, and phenotype is complemented by re-introduction of wild-type allele; Figure S3: Infection assay of xylanase mutants in FB1 × FB2 strains; Figure S4: Stress and cell wall integrity assays for xylanase defective mutants; Figure S5: Lack of xyn1 affects cell length in both CL13 and SG200 backgrounds. Table S1: List of primers used in this study.
