# Supplementary material for: Ustilago maydis Secreted Endo-Xylanases Are Involved in Fungal Filamentation and Proliferation on and Inside Plants
Source: J Fungi (Basel). 2021 Dec 15;7(12):1081. doi: 10.3390/jof7121081 (PMC8706147; doi:10.3390/jof7121081)
Supplement: Supplementary file 1 [file jof-07-01081-s001.zip › SI.pdf]

Supplementary Table S1. List of primers used in this study.

Name	Sequence (5′ – 3′)	Use
xyn1KO5-1	TATCACAGACGCCACGCTCG	*xyn1* deletion
xyn1KO5-2	CACGGCCTGAGTGGCCGATCACAACACAAGAG AGGAGGAGG	
xyn1KO3-1	GTGGCCATCTAGGCCTTCGTGATTGAAGATGCG TG	
xyn1KO3-2	CAAGTGTGAGTTGGGAGTGACG	
xyn1CO5_fwd	ACAGACACGAGAAGCAGGAGC	*xyn1* deletion validation
Nat2_rev	TGTACGCATGTAACATTATACTGAAAACCT	
Nat1_fwd	TGGCTGCTGATCACAGCAAGTCAGATT	
xyn1CO3_rev	AGGTGAAAGGTGAAATGTGC	
xyn1KOint-1	TTCGTCACCAACAAGATGTGC	
xyn1KOint-2	CCCACATGGATACACCAAAGG	
xyn2KO5-1	TAATCTGGCAGGGAGCTACG	*xyn2* deletion
xyn2KO5-2	CACGGCCTGAGTGGCCCCAGTGACCATTGCGTT TGC	
xyn2KO3-1	GTGGCCATCTAGGCCTTTGGACGATGCTGAGAA AGG	
xyn2KO3-2	TGCGAAGCAAAGAAAGACTACG	
xyn2CO5_fwd	CAAGCCTAACAAAGCTACCACG	*xyn2* deletion validation
Gen1_rev	TCTTCTGAGCGGGACTCTGG	
Gen2_fwd	GTACGGGTACATCGGATCTGC	
xyn2CO3_rev	AAGTGTAAAGCCCACAACGAGG	
xyn2KOint-1	ATACGCTCGTCTGGCACTCG	
xyn2KOint-2	CAGTTCTTCTGGTTGAGGCAGG	
xyn11AKO5-1	TAACGATCTCAGCCTCATGG	*xyn11A* deletion
xyn11AKO5-2	CACGGCCTGAGTGGCCATGGAGTTCGGAGACTG GTTCG	
xyn11AKO3-1	GTGGCCATCTAGGCCTTACTAATCCGACGCTGA AGG	
xyn11AKO3-2	CTTGTACCTCAACGCACTCC	
xyn11ACO5_fwd	ACTTCAAGTATGACCAGCACG	*xyn11A* deletion validation
hyg1_rev	AAGTTTGCAGAACTCGCTGG	
hyg2_fwd	CGATGGCTGTCTAGAAGTACTGCGCGATAG	
xyn11ACO3_rev	ACATCGAGGCAGAACCAGTACC	
xyn11AKOint-1	TGAAGATTACAACCCAGGTCC	
xyn11AKOint-2	GAACAGGTGACTCGAAGTGC	
Probe_nat_fwd	AAAAGGGGGACGGATCTAGG	Nat probe for Southern Blot
Probe_nat_rev	ACTGGATGGGTCCTTCACC	
Probe_gen_fwd	TACCGTAAAGCACGAGGAAGC	Geneticin probe for Southern Blot
Probe_gen_rev	CTCGACGTTGTCACTGAAGC	
Probe_hyg_fwd	AAACTGTGATGGACGACACC	Hygromycin probe for Southern Blot
Probe_hyg_rev	GCTCTATTCCTTTGCCCTCG	
Pxyn1_fwd	AGCAACGAGTCGACATCT	*xyn1* complementation
XmaI_Pxyn1_rev	TATCCCGGGTTTGATGAAGAGAAGATA	
Pxyn2_fwd	ACACTATAGAACTCGAGCAGAGTCGGCAAGCA AAACCG	*xyn2* complementation
Pxyn2_rev	TGGTCTTCATTGTGGAGGTAGGCTCTAAG	
xyn2_fwd	TACCTCCACAATGAAGACCAACTTTCTCG	
xyn2_rev	TGAACGATCTGCAGCCGGGCTCAAGCTTGGTAC GAGTTG	
Pxyn11A_fwd	ACACTATAGAACTCGAGCAGGGGTGAGTTCGAT TATCG	*xyn11A* complementation

Pxyn11A_rev	CAAACTTCATCTTGAATGTTCGAAGAAGAG	
xyn11A_fwd	AACATTCAAGATGAAGTTTGCCACTGTC	
xyn11A_rev	TGAACGATCTGCAGCCGGGCTCAACCAGAGACGGACATC	
N_Sdh2_fwd	TCCTGTCTTTTCGGCAAGACTCTTCG	*Ip* locus integration validation
N_pDL51_otef_rev	TGGTGCACTCTCAGTACAATCTGC	
Amp1_fwd	TTCTGTGACTGGTGAGTACTCAACC	
N_Sdh2_rev	TAAGTGACGATTGCGAGTTCTCTTGG	
BamHI_xyn2_fwd	ACGGGATCCATGAAGACCAACTTTCTCG	*xyn2* gfp tagging
NcoI_xyn2_rev	TCACCATGGCTCCAGCTTGGTACGAGTTGAGAGTGC	
BamHI_xyn11A_fwd	ACGGGATCCATGAAGTTTGCCACTGTCC	*xyn11A* gfp tagging
NcoI_xyn11A_rev	TCACCATGGCTCCACCAGAGACGGACATCG	
BamHI_xyn3_fwd	ACGGGATCCATGCCCGCACATCCTCATTTAGG	*xyn3* gfp tagging
NcoI_xyn3_rev	TCACCATGGCCTTCGGATCGAGCTTGTGTTTGAC	
SacII_xyn1_fwd	ATACCGCGGATGGTGAGCTCTAAGCTCGCCTTC	*xyn1* mCherry-HA tagging
XbaI_xyn1_rev	TATTCTAGACTTGCGACGTCGACGGTACGCCATG	
SacII_xyn2_fwd	TTACCGCGGATGAAGACCAACTTTCTCG	*xyn2* mCherry-HA tagging
NcoI-RSIATA_xyn2_rev	ATACCATGGCGGTGGCGATCGAGCGTTTAGCTTGGTACGAGTTG	
SacII_xyn11A_fwd	TTACCGCGGATGAAGTTTGCCACTGTCCTTGC	*xyn11A* mCherry-HA tagging
NcoI-RSIATA_xyn11A_rev	ATTATACCATGGCGGTGGCGATCGAGCGTCCACCAGAGACGGACATCGAG	
ppi_qPCR_fwd	ACATCGTCAAGGCTATCG	Biomass quantification
ppi_qPCR_rev	AAAGAACACCGGACTTGG	
gapdh_qPCR_fwd	CTTCGGCATTGTTGAGGGTTTG	
gapdh_qPCR_rev	TCCTTGGCTGAGGGTCCGTC	

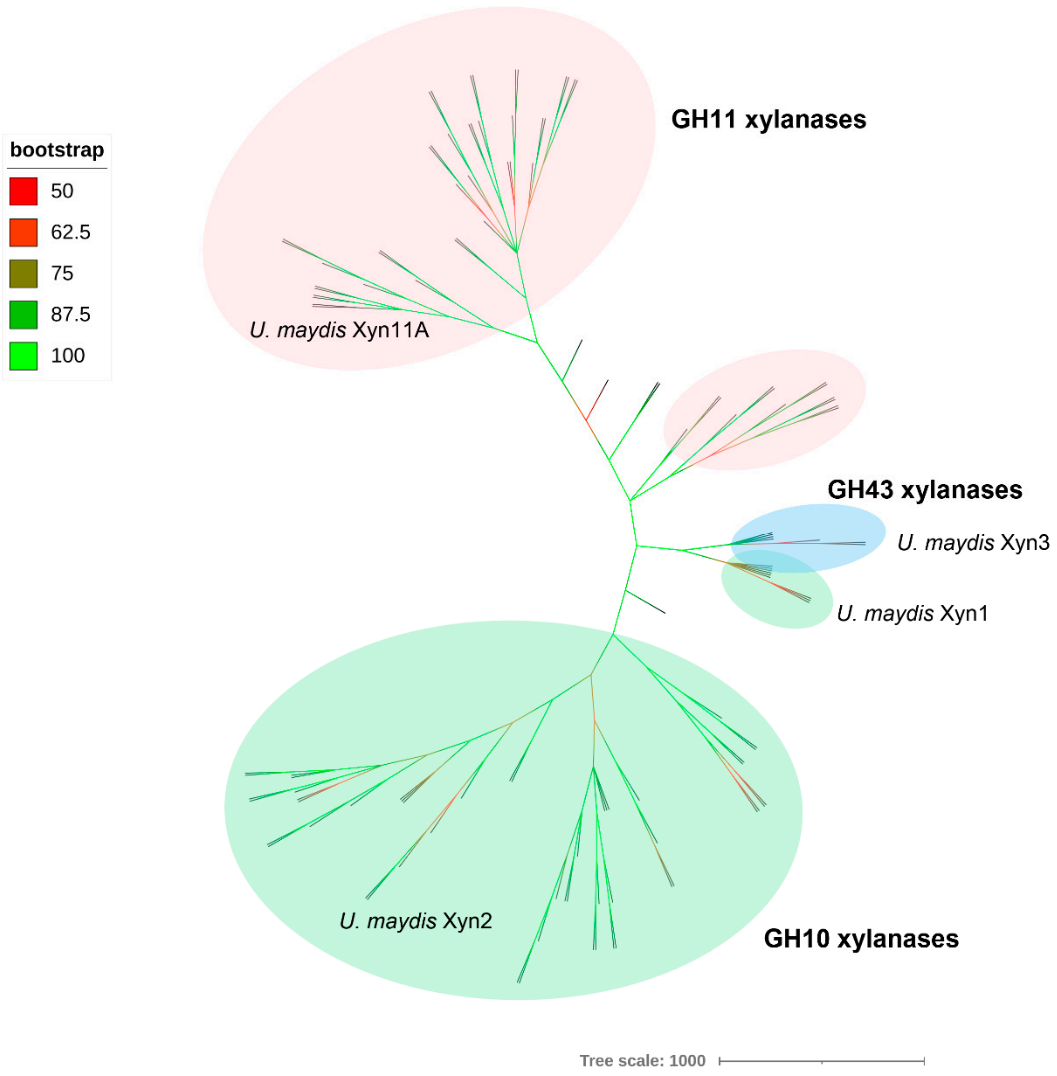

Figure S1. Fungal xylanases phylogenetic tree. An unrooted phylogenetic tree of xylanases from different Basidiomycetes and Ascomycetes plant pathogens was built (see Material and Methods for details). The tree was generated by applying the Neighbor-Join algorithm and the Jukes-Cantor genetic distance model to the 126 selected xylanase sequences previously aligned by Geneious alignment (Geneious Prime 2019.2.1). Xylanases from *U. maydis* Xyn1, Xyn2, Xyn11A and Xyn3 are indicated near to their nodes. Glycoside Hydrolase (GH) xylanases families are color-coded: green for GH10, red for GH11 and blue for GH43. Bootstraps are indicated in the branches with color gradient from red (50) to green (100).

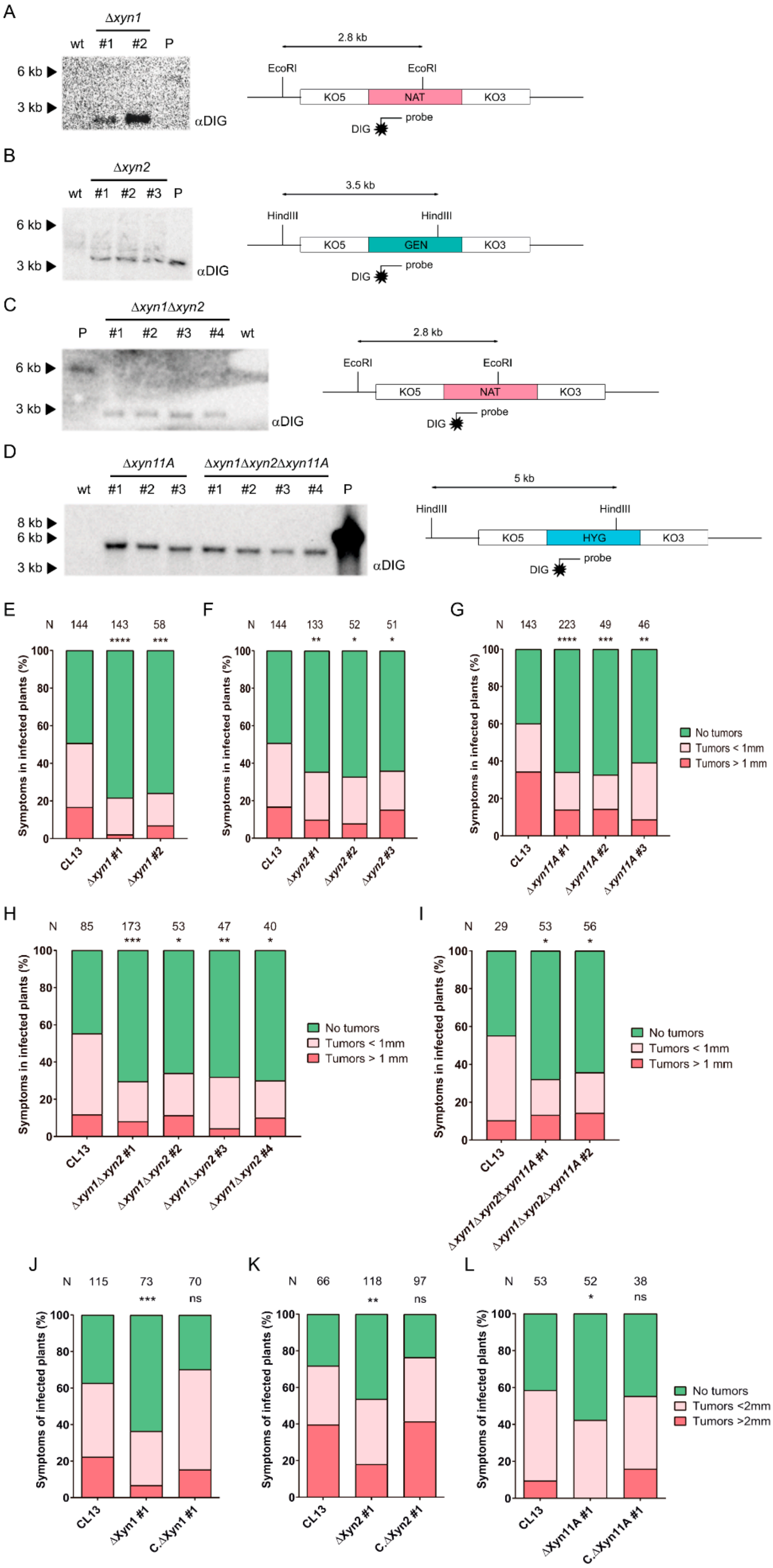

Figure S2. Xylanases mutant clones contain a single cassette integration, have similar virulence defects and are complemented with endogenous loci. (A-D) Southern blots of independent clones of *Δxyn1* **(A)**, *Δxyn2* **(B)**, *Δxyn1Δxyn2* **(C)**, *Δxyn11A* and *Δxyn1Δxyn2Δxyn11A* mutants **(D)** were performed in CL13

background. Representation of the binding site for each probe in the resistance markers (nourseothricin – NAT, geneticin – GEN, hygromycin – HYG) and the expected size of the band is represented in each panel. DNA from *wild-type* CL13 strain (wt) and each plasmid (P) containing xylanase deletion cassette was used as negative and positive controls, respectively. **(E-I)** Quantification of symptoms 14 days after infecting plants with independent clones of *Δxyn1* **(E)**, *Δxyn2* **(F)**, *Δxyn11A* **(G)**, *Δxyn1Δxyn2* **(H)** and *Δxyn1Δxyn2Δxyn11A* mutants **(I)**. **(J-L)** Quantification of symptoms 14 days after infecting plants with xylanases mutants complemented with the corresponding xylanase gene. Total number of infected plants is indicated above each column. At least two biological replicates were analyzed. Mann-Whitney statistical test was performed for each mutant versus the CL13 *wild-type* strain (ns for non-statistically significant; * for p-value < 0.05; ** for p-value < 0.01; *** for p-value < 0.005; **** for p-value < 0.001).

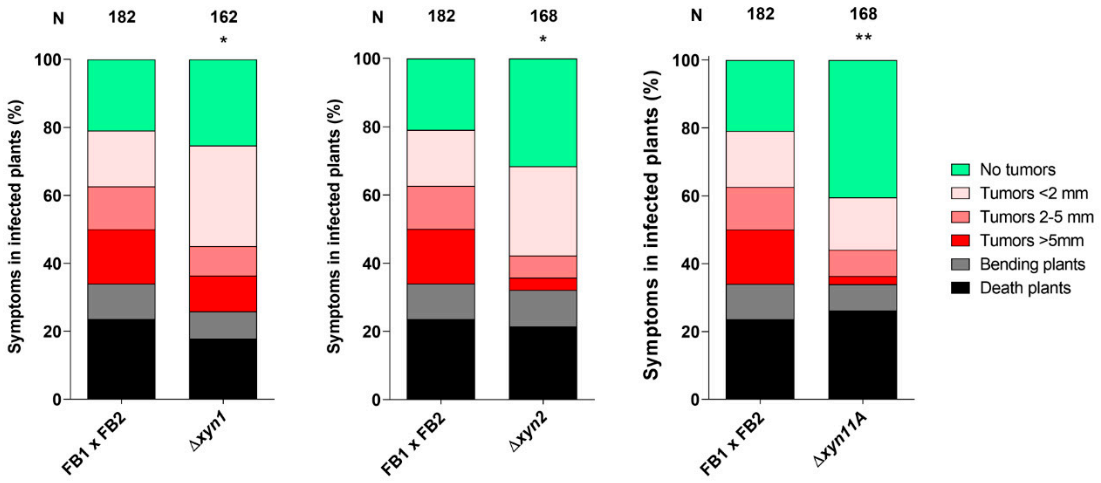

Figure S3. Infection assay for xylanases mutants in FB1 x FB2 strains. Quantification of plant symptoms infected with the indicated strains 14 days post infection. Total number of infected plants, corresponding to six biological replicates, is indicated above each column. The Mann-Whitney statistical test was performed for each mutant versus *wild-type* FB1 x FB2 strains (* for p-value < 0.05; ** for p-value < 0.01).

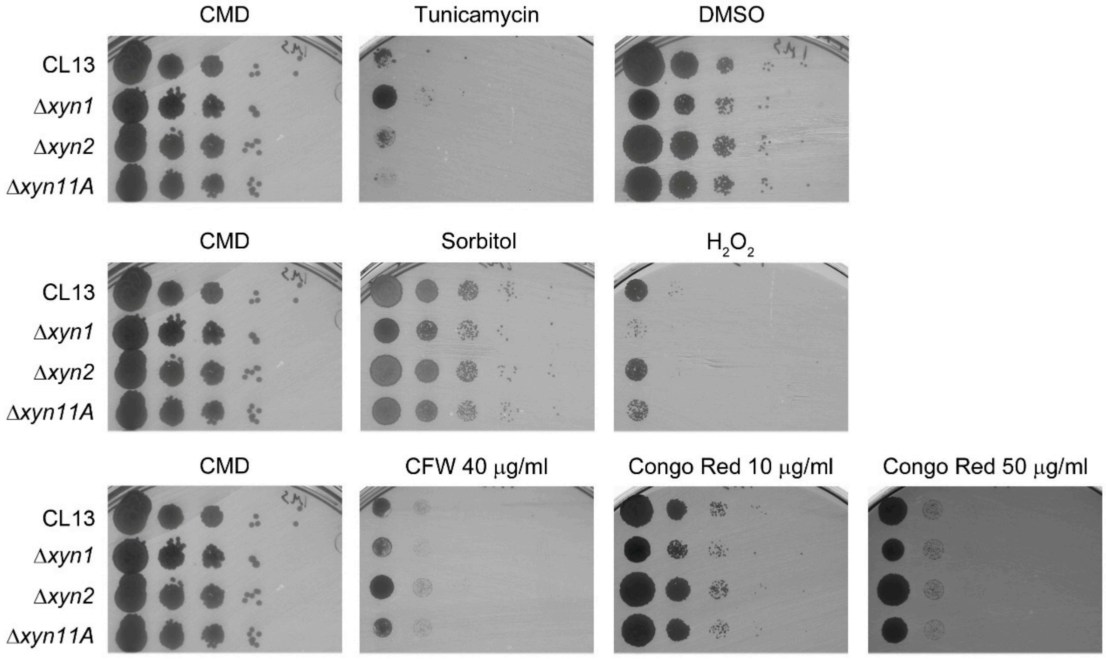

Figure S4. Stress and cell wall integrity assays for xylanases defective mutants. ER stress (Tunicamycin), osmotic stress (Sorbitol), oxidative stress (H_2O_2) and cell wall integrity (CFW and Congo Red) assays were

performed in rich media supplemented with 2% D-glucose (CMD) and 1 μg/ml Tunicamycin, 2% DMSO as tunicamycin solvent control, 1M Sorbitol, 1.5 mM H_2O_2, 50 μg/ml calcofluor white (CFW), and 10 and 50 μg/ml Congo Red.

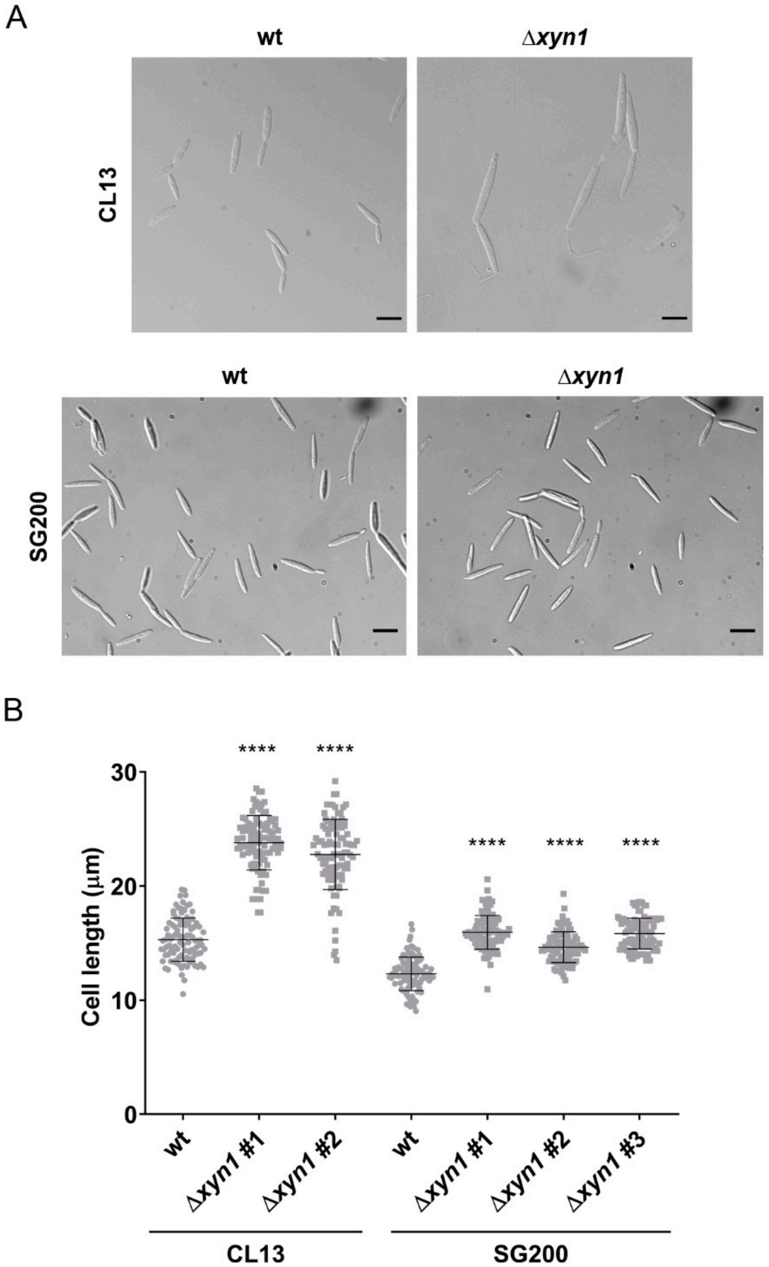

Figure S5. Lack of *xyn1* affects cell length in both CL13 and SG200 backgrounds. A) Cells were visualized by differential interference contrast (DIC) microscopy to analyze cellular morphology. Scale bars represent 10 μm. **B)** The lengths of CL13 *wild-type*, CL13 *Δxyn1* (two independent clones), SG200 *wild-type* and SG200 *Δxyn1* (three independent clones) strains were measured in rich media cultures at exponential phase. Quantification was done for 100 cells each from two biological replicates. T-test statistical analysis comparing each mutant versus the corresponding *wild-type* was performed (**** for p-value < 0.001).
